# Summary of Energy Informatics.Academy Conference 2022

**DOI:** 10.1186/s42162-022-00220-9

**Published:** 2022-12-21

**Authors:** Zheng Ma, Birte Holst Jørgensen, Guangchao Chen, Henrik Madsen, Hongbo Duan, Luiz Carlos Pereira da Silva, Bo Nørregaard Jørgensen

**Affiliations:** 1 SDU Center for Energy Informatics, The Maersk Mc-Kinney Moller Institute, University of Southern Denmark, Odense, Denmark; 2 Department of Wind Energy, Society, Market and Policy, Technical University of Denmark, Lyngby, Denmark; 3 College of Materials Sciences and Opto-Electronic Technology, University of Chinese academy of sciences, Beijing, China; 4 Department of Applied Mathematics and Computer Science, Technical University of Denmark, Lyngby, Denmark; 5 School of Economics & Management, University of Chinese Academy of Sciences, Beijing, China; 6 Department of Systems and Energy, University of Campinas (UN/ICAMP), Campinas, Brazil

The Energy Informatics.Academy Conference 2022 (EI.A 2022) (EnergyInformatics.Academy 2022) has collected great contributions from researchers and practitioners in various scientific, technological, engineering and social fields to disseminate original research on the application of digital technology and information management theory and practice to facilitate the global transition towards sustainable and resilient energy systems.

With the whole technical program committee’s effort, in total thirty-two (32) high-quality papers (including full papers and short papers) are accepted and presented at the conference. The thirty-two papers cover 4 important aspects of the energy informatics domain (shown in Table 1):

- Simulation and modeling in energy
- Software and applications in energy
- Big data and AI in energy
- Energy informatics projects and analysis

**Table 1:** Themes of the accepted and presented papers from Energy Informatics.Academy Conference 2022 (EI.A 2022)

Theme	Paper title
Simulation and modeling in energy	A Comparison Study of Co-simulation Frameworks for Multi-Energy Systems: The Scalability Problem
	Agent-based modeling (ABM) for urban neighborhood energy systems: Literature review and proposal for an all integrative ABM approach
	An agent-based modelling framework for the simulation of large-scale consumer participation in electricity market ecosystems
	Framework for Dimensioning Battery Energy Storage Systems with Applied Multi-tasking Strategies in Microgrids
	Simulation of a Cellular Energy System including Hierarchies and Neighborhoods
	A Hierarchical and Modular Agent-Oriented Framework for Power Systems Co-Simulations
	An Adapter-Based Architecture for Evaluating Candidate Solutions in Energy System Scheduling
	Automatic Process Monitoring in a District Heating Substation Utilizing a Contextual Shewhart Chart
Software and applications in energy	SGLSim: Tool for Smart Glazing Energy Performance Analysis
	Open-access Tools for the Modelling and Simulation of Electricity Markets
	Non-Intrusive Load Monitoring techniques for the disaggregation of ON/OFF appliances
	Design of Data Management Service Platform for Intelligent Electric Vehicle Charging Controller—Multi-charger Model
	Design of an intelligent trading platform for flexibility potentials of private households in the low-voltage grid
	Can we benefit from game engines to develop digital twins for planning the deployment of photovoltaics?
	Probabilistic FlexOffers in Residential Heat Pumps Considering Uncertain Weather Forecast
	Potentials of game engines for wind power digital twin development: an investigation of the Unreal engine
Big data and AI in energy	Peer-to-Peer Energy Trading Optimization in Energy Communities using Multi-Agent Deep Reinforcement Learning
	Investigation on Air Conditioning Load Patterns and Electricity Consumption of Typical Residential Buildings in Tropical Wet and Dry Climate in India
	Residential Electricity current and appliance dataset for AC-event detection from Indian Dwellings
	An Implementation of Long Short-Term Memory on Electricity Load Forecasting: Comparison of Multiple Scalers
	Anomaly detection in quasi-periodic energy consumption data series: a comparison of algorithms
	Revealing interactions between HVDC cross-area flows and frequency stability with explainable AI
	Recursive training based Physics Inspired Neural Network for Electric Water Heater modeling
	Evaluation of Neural Networks for Residential Load Forecasting and the Impact of Systematic Feature Identification
	Identification of natural disaster impacted electricity load profiles with k means clustering algorithm
Energy informatics projects and analysis	Can electric vehicles be an alternative for traditional fossil-fuel cars with the help of renewable energy sources towards energy sustainability achievement?
	Impact of the COVID-19 on residential energy consumption of Hyderabad, India
	A probabilistic approach to reliability analysis of district heating networks incorporation censoring: A report of implementation experiences
	Application of Energy Informatics in Danish Research Projects
	CSTEP-driven business opportunity identification method with a case study of energy use in industrial processes
	Survey data on university students’ experience of energy control, indoor comfort, and energy flexibility in campus buildings
	CELSIUS: an international project providing integrated, systematic, Cost-effective large-scale IoT solutions for improving energy efficiency of medium- and large-sized buildings

The paper presentations are recorded and available via EnergyInformatics.Academy YouTube channel (EnergyInformatics.Academy 2022; EnergyInformatics.Academy 2022).

Six keynote speakers shared their great experience and knowledge with six speeches and a panel discussion (shown in Table 2). The presentation slides are available via EnergyInformatics.Academy webpage (EnergyInformatics.Academy 2022).

**Table 2:** Six keynote speeches at the Energy Informatics.Academy(EI.A) Asia 2021 conference

Keynote speech title	Speakers	Affiliation
Digital Twin modelling of Energy Systems	Bo Nørregaard Jørgensen	University of Southern Denmark
The importance of digitization and architectural thinking to drive long term sustainability	Brian Skov Lykke Rasmussen	IBM
FlexOffers: Towards an Open Standard for Energy Flexibility	Torben Bach Pedersen	Aalborg University
Data-driven methods for Smart Energy Systems	Henrik Madsen	Technical University of Denmark
Data-driven innovation for the green transition	Søren Skov Bording	Center Denmark
Panel session-Accelerating the digital transformation in energy systems		

The EI.A 2022 were co-hosted with the Innovation Festival at Dandy Business Park and the industrial and PhD summer school in Energy Informatics. The EI.A 2022 could not success without the following organizations’ support:

- Springer Open Journal Energy Informatics
- Sino-Danish Center
- Dandy Business Park
- SDU Center for Energy Informatics
- Energy Informatics.Academy
- European Union Social Fund
- UNITEN Institute of Informatics and Computing in Energy

## Data Availability

Not applicable.

## Abbreviation

EI.A: Energy Informatics.Academy
